# Post‐Hemodialysis Flow‐Dependent Hepatic Function Impairment in Individuals With End Stage Kidney Disease and Chronic Inflammation

**DOI:** 10.1111/hdi.70016

**Published:** 2025-08-14

**Authors:** Oscar Swift, Bobby V. M. Dasari, Malcolm A. Finkelman, Yonglong Zhang, Sivakumar Sridharan, Enric Vilar, Ken Farrington

**Affiliations:** ^1^ School of Life and Medical Science University of Hertfordshire Hatfield Hertfordshire UK; ^2^ Department of Renal Medicine Lister Hospital, East and North Hertfordshire NHS Trust Stevenage Hertfordshire UK; ^3^ Department of Hepatopancreatobiliary and Liver Transplant Surgery Queen Elizabeth Hospital, University Hospitals Birmingham NHS Foundation Trust Birmingham West Midlands UK; ^4^ Associates of Cape Cod Inc. East Falmouth Massachusetts USA

**Keywords:** beta‐D‐glucan, chronic inflammation, hepatic function

## Abstract

**Introduction:**

The liver plays an important role to prevent translocation of gut‐derived toxins from the portal to the systemic circulation. Chronic inflammation is common in patients receiving hemodialysis, and increased gut permeability to microbial material has been implicated in its pathogenesis. This study sought to establish if flow‐dependent hepatic function was impaired in chronically inflamed individuals treated with hemodialysis.

**Methods:**

Fifty adults receiving outpatient hemodialysis were recruited. Subjects with known liver or gastrointestinal disease, acute inflammation, and hemodynamic instability during hemodialysis were excluded.

Participants were divided into two groups (*n* = 25): individuals with chronic inflammation (defined as a median high‐sensitivity C‐reactive protein (hs‐CRP) ≥ 5 mg/dL over the preceding 3 months) with no apparent cause and a noninflamed group.

Flow‐dependent hepatic function (defined as a composite of hepatic perfusion, hepatocyte clearance and biliary excretion) was assessed following hemodialysis by indocyanine green clearance to derive: (1) indocyanine green‐plasma disappearance rate and (2) indocyanine green‐retention after 15 min. Serum beta‐D‐glucan levels pre‐ and post‐hemodialysis were measured as surrogate markers of gastrointestinal permeability.

**Findings:**

Indocyanine green‐plasma disappearance rate was reduced in the inflamed group versus the noninflamed group (19.4 (8.7)%/min vs. 23.8 (14.4)%/min; *p* = 0.02). Indocyanine green‐retention after 15 min was higher in the inflamed group (5.4 (6.8)% vs. 2.9 (5.0)%; *p* = 0.02). Noninvasive hepatic fibrosis and steatosis assessments were similar in both groups. Pre‐hemodialysis beta‐D‐glucan levels were similar (63 (42) pg/ml vs. 49 (11) pg/ml; *p* = 0.13), whereas post‐hemodialysis beta‐D‐glucan levels were higher in the inflamed group (82 (48) pg/ml vs. 58 (27) pg/ml; *p* < 0.001), and in those with flow‐dependent hepatic impairment (72 (45) vs. 55 (32) pg/ml; *p* = 0.004). In linear regression analysis, indocyanine green‐retention after 15 min and post‐hemodialysis beta‐D‐glucan levels were independent predictors of median hs‐CRP, explaining 21% of the variation.

**Discussion:**

Individuals with otherwise unexplained inflammation had impaired hepatic function post‐hemodialysis and higher post‐hemodialysis beta‐D‐glucan levels. These findings are compatible with the notion that impaired hepatic gut‐derived toxin removal propagates chronic inflammation in hemodialysis.

## 1 Introduction

Chronic inflammation is common in end‐stage kidney disease (ESKD) and is often unexplained by any cause other than kidney failure itself or the provision of renal replacement therapy. This can be termed ESKD‐associated chronic inflammation. Such inflammation is linked to accelerated cardiovascular disease, poor clinical outcomes, and significant symptom burden, and is particularly prevalent in people receiving hemodialysis treatment [1, 2, 3]. The pathophysiology of ESKD‐associated chronic inflammation is complex and poorly understood. Multiple factors have been implicated, including uraemic toxin retention, salt and water overload, dialyzer membranes, oxidative stress, and immune dysfunction [4]. Evidence is emerging for the role of microbial fragment translocation from the gut lumen into the systemic circulation as a significant factor in ESKD‐associated chronic inflammation development [5]. Increased gut permeability in ESKD may be a direct effect of uraemic toxins on gut barrier integrity [6], exacerbated by splanchnic hypoxia and subclinical bowel ischemia as a consequence of rapid ultrafiltration rates during hemodialysis [7, 8, 9].

Liver disease is associated with increased gut permeability and endotoxemia, even prior to the development of cirrhosis [10]. In normal circumstances, the liver, in particular resident hepatic macrophages (Kupffer cells), plays a major role in preventing the passage of gut‐derived material from the portal into the systemic circulation [11]. Hence, it may be that two co‐existing conditions are required to allow gut‐derived toxins to penetrate into the systemic circulation and stimulate an inflammatory response in ESKD: (1) increased gut permeability and (2) hepatic dysfunction. Both of these conditions are present with advanced liver disease, but in individuals with ESKD‐associated chronic inflammation with evidence of increased gastrointestinal permeability, it is unclear if there is evidence of a “second hit” of concomitant hepatic dysfunction [12].

Chronic liver disease is prevalent in ESKD, especially in areas with hepatitis B and C prevalence [13, 14]. Five percentage of the dialysis population globally have cirrhosis [15]. However, even in hemodialysis patients without established chronic liver disease, changes in both hepatic perfusion and function occur during a hemodialysis session [16, 17]. It is therefore important to establish whether hemodialysis‐related changes in hepatic function are linked to ESKD‐associated chronic inflammation and markers of gut permeability.

Flow‐dependent hepatic function can be measured by indocyanine green clearance. Indocyanine green is a highly protein‐bound, fluorescent dye exclusively cleared by the liver [18]. Indocyanine green clearance is validated for measuring flow‐dependent hepatic function (defined as a composite of hepatic perfusion, hepatocyte clearance and biliary excretion) in hepatological and critical care settings [19, 20, 21].

The gold standard method to investigate gut permeability relies on urinary saccharide concentration measurement following ingestion of several probes. These levels are critically dependent on kidney function [22]; so this method is untenable in ESKD without additional development. Measurement of serum levels of (1‐3)‐beta‐D glucan (beta‐D‐glucan) is a potential alternative method to assess gut permeability [23]. Beta‐D‐glucan comprises a range of glucose polymers found in dietary plant material, as well as fungal and bacterial cell walls [24]. Beta‐D‐glucan predominantly undergoes reticuloendothelial (primarily hepatic) clearance [25]. Elevated levels of beta‐D‐glucan in advanced kidney disease [26] may reflect systemic translocation of microbial structures from the gut. Beta‐D‐glucan can activate the Limulus Amoebocyte Lysate assay used to detect endotoxin, and can induce false‐positive results [27]. Fecal calprotectin reflects gastrointestinal inflammation and is another potential marker of gut barrier impairment in ESKD unaffected by native kidney function.

We hypothesized that in individuals treated with hemodialysis without evidence of chronic liver disease, those with ESKD‐associated chronic inflammation would have greater post‐hemodialysis hepatic dysfunction and higher serum beta‐D‐glucan levels than noninflamed individuals. Such findings would be consistent with the theory that gut hyperpermeability in combination with hepatic dysfunction contributes to ESKD‐associated chronic inflammation.

## 2 Materials and Methods

### 2.1 Study Setting and Regulatory Requirements

Participants were recruited from four hemodialysis units managed by East and North Hertfordshire National Health Service (NHS) Trust, United Kingdom, following provision of informed, written consent. The study was performed in accordance with approved protocols, the Declaration of Helsinki, and applicable regulatory requirements. Ethical approval was obtained from the NHS South Central–Hampshire B Research Ethics Committee (Reference: 21/SC/0140). The study was prospectively registered and adopted onto the National Institute of Health and Care Research Clinical Research Portfolio (Identification: 49132).

### 2.2 Study Design

#### 2.2.1 Inclusion Criteria

All participants were adults (age ≥ 18) receiving maintenance outpatient hemodialysis (either high‐flux hemodialysis or online hemodiafiltration) for at least 3 months.

Two groups were recruited: 25 individuals with ESKD‐associated chronic inflammation (defined as a median baseline high‐sensitivity C‐reactive protein (hs‐CRP) ≥ 5 mg/L over the previous 3 months) and 25 individuals without chronic inflammation (a median baseline hs‐CRP < 5 mg/L over the previous 3 months). A hs‐CRP cut off ≥ 5 mg/L was chosen as the normal range for the assay was 0–5 mg/L, and additionally because other studies had defined chronic inflammation in ESKD as an hs‐CRP of ≥ 5 mg/L [28, 29].

#### 2.2.2 Exclusion Criteria

Exclusion criteria included: a history of adverse reactions to indocyanine green or iodine, thyroid disease, underlying liver or active gastrointestinal disease, active inflammation within the previous 3 months due to infection, autoimmune disease or malignancy, documented evidence of peripheral vascular disease, and hemodynamic instability during hemodialysis. All available clinical, laboratory, microbiology, radiology, histopathology, and endoscopy records for participants were reviewed, accompanied by a comprehensive clinical history and examination to evaluate inclusion and exclusion criteria.

### 2.3 Study Procedures in Relation to Hemodialysis

All participants received hemodialysis according to their usual prescription. The following procedures were carried out in relation to the hemodialysis session (and are described below):

#### 2.3.1 Pre‐Hemodialysis

1. Baseline demographic, clinical, and hemodialysis‐related information was recorded.
2. Serum samples were collected for routine biochemistry and hematology.
3. A fresh stool sample was provided for fecal calprotectin analysis.
4. Patient reported outcomes including the Standardized Outcomes in Nephrology (SONG)‐hemodialysis fatigue instrument [30] and the eight item Patient Health Questionnaire depression scale (PHQ‐8) [31] were obtained.

#### 2.3.2 Pre‐ and Post‐Hemodialysis

1. Clinical assessment of volume status was performed; supplemented, pre‐hemodialysis, by body composition analysis (Body Composition Monitor, Fresenius Medical Care, Germany).
2. Serum levels of beta‐D‐glucan with a paired hematocrit level were obtained.
3. Assessment of liver stiffness and steatosis by vibration‐controlled transient elastography and controlled attenuation parametography using a FibroScan 530 device (FibroScan, Echosens, France) was performed.

#### 2.3.3 Post‐Hemodialysis

1. Assessment of flow‐dependent hepatic function was measured by indocyanine green clearance.
2. Dialysis‐related parameters including intradialytic weight change, ultrafiltration rate, and volume were recorded.

### 2.4 Methodology

#### 2.4.1 Beta‐D‐Glucan, Fecal Calprotectin and Baseline Biochemical and Hematology Assessment

Beta‐D‐glucan levels were measured following a three‐hour fast using the Fungitell assay (Associates of Cape Cod Inc., USA) [32]. Participants were allowed to eat following the initial pre‐dialysis beta‐D‐glucan measurement but were fasted again for 3 h prior to the post‐dialysis measurement.

Post‐hemodialysis beta‐D‐glucan levels were adjusted for changes in hemoconcentration for the plasma compartment to mitigate against artefactual changes that may occur secondary to ultrafiltration. Fecal calprotectin levels were measured using a particle enhanced turbidimetric immunoassay (BÜHLMANN Laboratories AG, Switzerland). Routine biochemistry and hematological parameters were measured using standard local laboratory assays.

#### 2.4.2 Body Composition Analysis

Study participants underwent body composition assessment using standard tetrapolar techniques (Body Composition Monitor, Fresenius Medical Care, Germany), with electrodes placed at both wrists and ankles after subjects had been supine for 10 min [33].

#### 2.4.3 Dialytic Treatment

All study participants received either high‐flux hemodialysis (20%) or hemodiafiltration (80%) in‐center. The majority of participants dialyzed thrice weekly (88%), with a mean session time of 230 ± 17 min. Apart from participants with significant residual kidney function treated with incremental hemodialysis and participants requiring an augmented hemodialysis schedule, an equilibrated Kt/V of 1.2 for thrice weekly participants was targeted. Microbiological contaminant levels of hemodialysis fluid at all sites were < 0.1 colony forming units per milliliter, and endotoxin concentrations were < 0.03 international units per milliliter. Participants were treated with either a polysulfone membrane (FX Cordiax, Fresenius Medical Care, Germany) (*n* = 42), a heparin‐grafted AN69 ST membrane (Evodial, Baxter, USA) (*n* = 4) or a cellulose triacetate membrane (Sureflux, Nipro, Japan) (*n* = 4). Sessional ultrafiltration volume was prescribed following clinical assessment.

#### 2.4.4 Indocyanine Green Clearance

0.25 mg/kg of indocyanine green (Verdye, Kimal, UK) was administered intravenously as a bolus to participants immediately following completion of a hemodialysis session. Participants were fasted for 3 h prior to indocyanine green administration to limit post‐prandial changes in hepatic blood flow. Indocyanine green clearance was assessed continuously with a noninvasive technique using transcutaneous pulse spectrophotometry on a finger clip device (LiMON, PULSION Medical Systems, Germany) [34]. Optical light at two wavelengths: 805 nm (maximal indocyanine green absorption) and 890 nm (minimal indocyanine green absorption) measured real‐time changes in arterial indocyanine green blood concentrations based on differences in absorbance between oxyhemoglobin and indocyanine green [35]. This data was used to calculate indocyanine green‐plasma disappearance rate and indocyanine green‐retention after 15 min. In participants with an arteriovenous fistula, measurements were performed in the contralateral arm to the hemodialysis access to avoid spurious results.

#### 2.4.5 Assessment of Liver Stiffness and Steatosis

Vibration‐controlled transient elastography and controlled attenuation parametography were performed to evaluate liver stiffness and hepatic steatosis, respectively. Participants were scanned both before and after hemodialysis following a three‐hour fast prior to each scan. Only scores obtained from a minimum of 10 consecutive measurements with an interquartile range of ≤ 30% were considered valid and used in analyses. Because of the potential effects of volume overload and hepatic blood flow changes during hemodialysis on liver stiffness measurements, results from the scan that obtained the lowest liver stiffness measurement (either pre‐hemodialysis or post‐hemodialysis) were used in the final analyses. A fibrosis‐4 (FIB‐4) score [36] was calculated as an additional screening tool for underlying, undiagnosed liver disease.

#### 2.4.6 Power Calculations

Power calculations were based on existing data indicating an indocyanine green‐plasma disappearance rate of 23.5% ± 2.4%/min in normal individuals [35] and 18.2% ± 5.2%/min in participants with ESKD [16]. Assuming similar intergroup differences between inflamed and noninflamed hemodialysis subgroups, a *t*‐test of a sample size of 16 per group yielded a study power of 95% and a type I (alpha) error probability of 5%. However, given the methodological differences between the study groups involved in the power calculations and those in this study, sample sizes were pragmatically increased to 25 per group.

#### 2.4.7 Statistical Analyses

Analyses were performed using IBM SPSS Statistics, Version 28 (IBM, USA). Continuous variables were tested for normality using the Shapiro–Wilk test. Parametric data are presented as mean ± standard deviation. Nonparametric data are presented as median (interquartile range). Comparison of two groups were performed with *t*‐test or Mann–Whitney U analyses according to distribution. Comparisons of continuous data between multiple groups used one‐way ANOVA or Kruskal‐Wallis tests. Proportions were compared using the Chi‐squared test. Two‐tailed *p* values < 0.05 were considered statistically significant. Receiver operating characteristic (ROC) analysis was used to define relationships between both indocyanine green‐plasma disappearance rate and indocyanine green‐retention after 15 min and chronic inflammation defined as median baseline hs‐CRP ≥ 5 mg/L. Linear and logistic regression models were constructed to examine contributions of indocyanine green clearance and beta‐D‐glucan levels to median hs‐CRP levels. Nonparametrically distributed variables were log transformed for inclusion.

## 3 Results

### 3.1 Demographics and Clinical Characteristics

Study population characteristics are presented in Table 1. Baseline age, comorbidity, dialysis vintage, and temperature, tunneled dialysis catheter use, volume status, ultrafiltration rate, and dialysis dose were similar in both groups. Participants in the chronically inflamed group had a higher body mass index (BMI), white cell count, and platelet count. The chronically inflamed group had less residual kidney function and was treated with higher rates of hemodiafiltration.

**TABLE 1:** Study population characteristics.

	Non‐inflamed (*n* = 25)	Inflamed (*n* = 25)	*p*
Clinical characteristics			
Age (years)	65 ± 14	61 ± 16	0.42
% male	56	76	0.14
% white (self‐reported ethnicity)	76	88	0.27
Body mass index (kg/m^2^)	27.8 (8.9)	33.3 (7.3)	**0.02**
Waist to height ratio^a^	0.63 ± 0.10	0.69 ± 0.10	0.06
Lean tissue index (kg/m^2^)^b^	13.5 ± 2.8	15.0 ± 4.2	0.20
Fat tissue index (kg/m^2^)^b^	15.1 ± 7.5	19.8 ± 9.5	0.08
Charlson comorbidity index	5.9 ± 2.8	5.3 ± 2.5	0.43
Diabetes mellitus (%)	36	40	0.77
Ischemic heart disease (%)	20	16	0.71
Cardiac failure (%)	16	16	1.00
Patient temperature (°C)	35.7 ± 0.7	35.8 ± 0.5	0.55
Pre‐dialysis systolic BP (mmHg)	150 ± 16	138 ± 27	0.07
Post‐dialysis systolic BP (mmHg)	140 ± 29	135 ± 21	0.49
Pre‐dialysis diastolic BP (mmHg)	75 ± 19	70 ± 15	0.27
Post‐dialysis diastolic BP (mmHg)	66 ± 11	67 ± 13	0.90
Dialysis‐related characteristics			
Dialysis vintage (months)	23 (23)	33 (42)	0.11
Dialysate temperature (°C)	36.0 (0.0)	36.0 (0.0)	0.60
% Tunneled dialysis catheter use	32	24	0.26
Residual urea clearance (ml/min)^c^	2.3 (2.5)	0.0 (2.0)	**0.03**
Extracellular: intracellular volume^b^	0.9 ± 0.1	0.9 ± 0.2	0.69
Pre‐dialysis excess volume (L)^b^	1.4 (1.9)	1.1 (3.3)	0.58
Pre‐dialysis excess volume (as % of total extracellular volume)	7.8 (10.0)	4.2 (15.4)	0.24
Hemodiafiltration treatment (%)	64	96	**0.005**
Dialysis session time (min)	225 (30)	240 (30)	0.42
Ultrafiltration rate (ml/kg/h)	4.8 ± 3.1	4.2 ± 2.9	0.50
Equilibrated Kt/V^d^	1.24 ± 0.28	1.16 ± 0.28	0.27
Dialysis membrane			
Polysulfone (%)	84	84	1.00
AN69 (%)	8	8	1.00
Cellulose triacetate (%)	8	8	1.00
Laboratory data			
Serum hs‐CRP (mg/L)	1.8 (2.8)	10.0 (8.6)	**< 0.001**
Serum creatinine (mg/dL)	8.0 ± 3.2	8.5 ± 2.0	0.49
Serum albumin (g/dL)	4.1 ± 0.3	3.9 ± 0.4	0.07
Hemoglobin (g/dL)	11.0 ± 0.9	10.7 ± 1.2	0.24
White cell count (×10^3^/μL)	6.3 ± 1.8	7.7 ± 2.5	**0.03**
Platelet count (×10^3^/μL)	195 ± 61	240 ± 64	**0.02**
Ferritin (μmol/L)	326 (486)	330 (375)	0.69
International normalized ratio (INR)	1.0 (0.0)	1.0 (0.0)	0.99
Serum bilirubin (mg/dL)	0.3 (0.2)	0.3 (0.1)	0.99
Alanine aminotransferase (units/L)	15 (9)	15 (7)	0.44
Alkaline phosphatase (units/L)	77 (35)	102 (60)	**0.001**
Gamma‐glutamyl transpeptidase (units/L)	18 (15)	31 (34)	**0.005**
Aspartate aminotransferase (units/L)	18 (8)	16 (5)	0.43
Serum cholesterol (mg/dL)	0.4 ± 0.1	0.4 ± 0.1	0.95
Serum triglyceride (mg/dL)	0.1 (0.1)	0.1 (0.1)	0.29

*Note:* Results for continuous variables presented as mean ± standard deviation or median (interquartile range) according to distribution. Conversion factors for units: serum creatinine in mg/dL to μmol/L, ×88.4; serum bilirubin in mg/dL to μmol/L, ×17.1. The bold values are those that have a *p*‐value of < 0.05 and thus are deemed statistically significant.

^a^ Waist circumference data available for 22 noninflamed and 24 inflamed participants.

^b^ Fat tissue index, extracellular: intracellular volume and excess volume data available for 22 noninflamed and 20 inflamed participants.

^c^ Residual urea clearance data available for 22 noninflamed and 19 inflamed participants.

^d^ Equilibrated Kt/V data available for 25 noninflamed and 24 inflamed participants.

### 3.2 Hepatic Function

Markers of synthetic hepatic function (serum albumin, bilirubin and international normalized ratio) and transaminases (alanine aminotransferase and aspartate aminotransferase) were similar in both groups (Table 1). Alkaline phosphatase and gamma‐glutamyl transpeptidase levels were both higher in the inflamed group.

Results of flow‐dependent hepatic function and assessments of hepatic steatosis and fibrosis are presented in Table 2. Indocyanine green clearance results were obtained for 47 participants (24 noninflamed and 23 inflamed); in three participants, there was insufficient perfusion to the finger to obtain accurate pulse spectrophotometry.

**TABLE 2:** Differences in hepatic function and noninvasive liver fibrosis and steatosis assessments.

	Non‐inflamed (*n* = 24)	Inflamed (*n* = 23)	*p*‐value
ICG‐PDR (%/min)^a^	23.8 (14.4)	19.4 (8.7)	**0.02**
ICG‐R15min (%)^a^	2.9 (5.0)	5.4 (6.8)	**0.02**
Liver stiffness measurement (kPa)^b^	4.8 (2.2)	4.2 (3.2)	0.69
Controlled attenuation parametography (dB/m)^b^	249 ± 69	257 ± 63	0.69
FIB‐4 score	1.42 (1.43)	1.00 (1.00)	**0.04**

*Note:* Results for continuous variables presented as mean ± standard deviation or median (interquartile range) according to distribution. The bold values are those that have a *p*‐value of < 0.05 and thus are deemed statistically significant.

^a^ Indocyanine green kinetics data available for 24 noninflamed and 23 inflamed participants.

^b^ Valid FibroScan data available for 25 inflamed and 24 non‐inflamed participants.

Indocyanine green clearance was significantly reduced at the end of hemodialysis in the inflamed group compared to the noninflamed group (indocyanine green‐plasma disappearance rate 19.4 (8.7)%/min vs. 23.8 (14.4)%/min; *p* = 0.02). Indocyanine green‐retention after 15 min was higher in the inflamed group (5.4 (6.8)% vs. 2.9 (5.0)%; *p* = 0.02). Differences in flow‐dependent function between inflamed and noninflamed groups were apparent despite similar liver stiffness (4.2 (3.2) kPa vs. 4.8 (2.2) kPa; *p* = 0.69) and hepatic steatosis measurements (257 ± 63 dB/m vs. 249 ± 69 dB/m; *p* = 0.69) (Table 2). FIB‐4 scores were higher in the noninflamed group compared to the inflamed group (1.42 (1.43) vs. 1.00 (1.00); *p* = 0.04). The use of high‐flux hemodialysis or hemodiafiltration did not have a significant impact on either indocyanine green‐plasma disappearance rate (23.7 (10.2)% vs. 20.7 (12.8)% (*p* = 0.28)) or indocyanine green‐retention after 15 min (2.9 (3.7)% vs. 4.5 (7.8)% (*p* = 0.30)).

**FIGURE 1:**
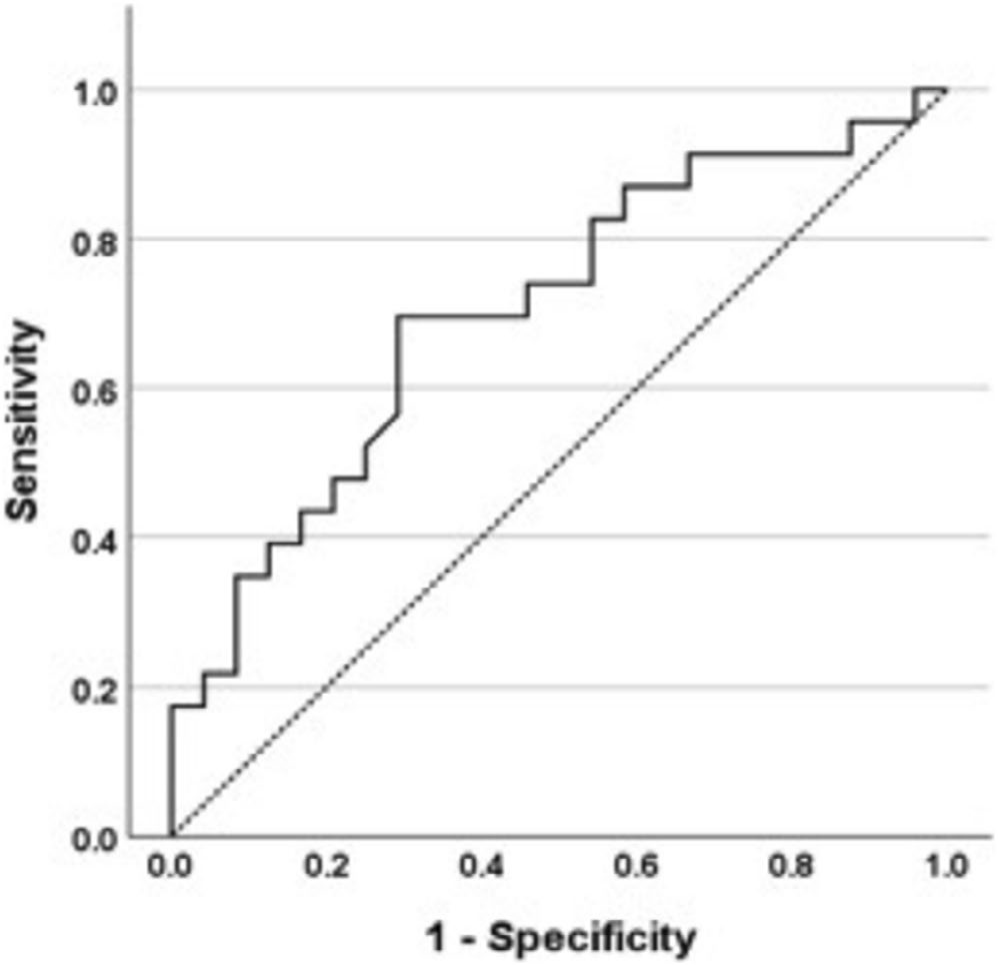
Receiver operator curve analysis for relationship of indocyanine green‐plasma disappearance rate and inflammation (hs‐CRP ≥ 5 mg/L). AUC, area under curve.

**FIGURE 2:**
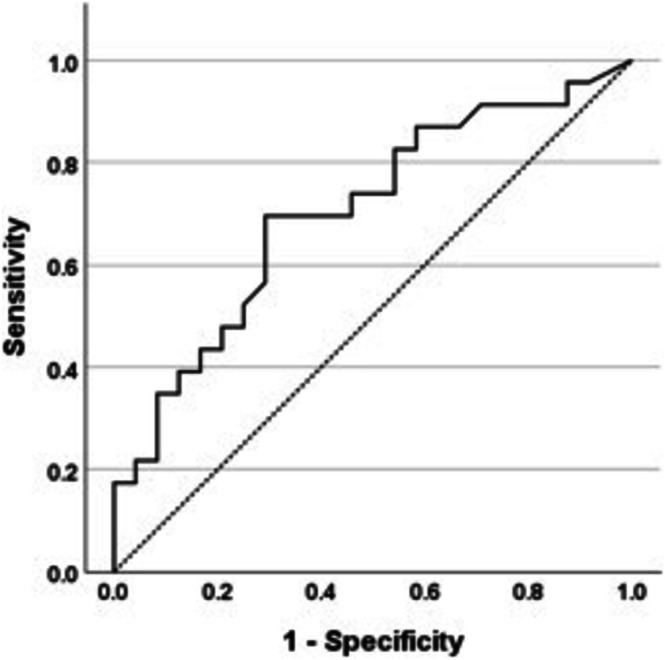
Receiver operator curve analysis for analysis of relationship of indocyanine green retention after 15 min and inflammation (hs‐CRP ≥ 5 mg/L). AUC, area under curve.

ROC curve analysis defined relationships between inflammation and indocyanine green clearance. Area under the curve (AUC) for indocyanine green‐plasma disappearance rate was 0.704 (Figure 1). The best cut‐off value for indocyanine green‐plasma disappearance rate was 21%/min (70% sensitivity and 71% specificity). 70% of inflamed participants had an indocyanine green‐plasma disappearance rate < 21%/min compared to 29% of noninflamed participants (*p* = 0.006). The AUC for indocyanine green‐retention after 15 min was 0.703 (Figure 2). The best cut‐off value for indocyanine green‐retention after 15 min was 7.5%. 40% of inflamed individuals had an indocyanine green‐retention after 15 min level > 7.5% compared with 13% of noninflamed participants (*p* = 0.04).

**FIGURE 3:**
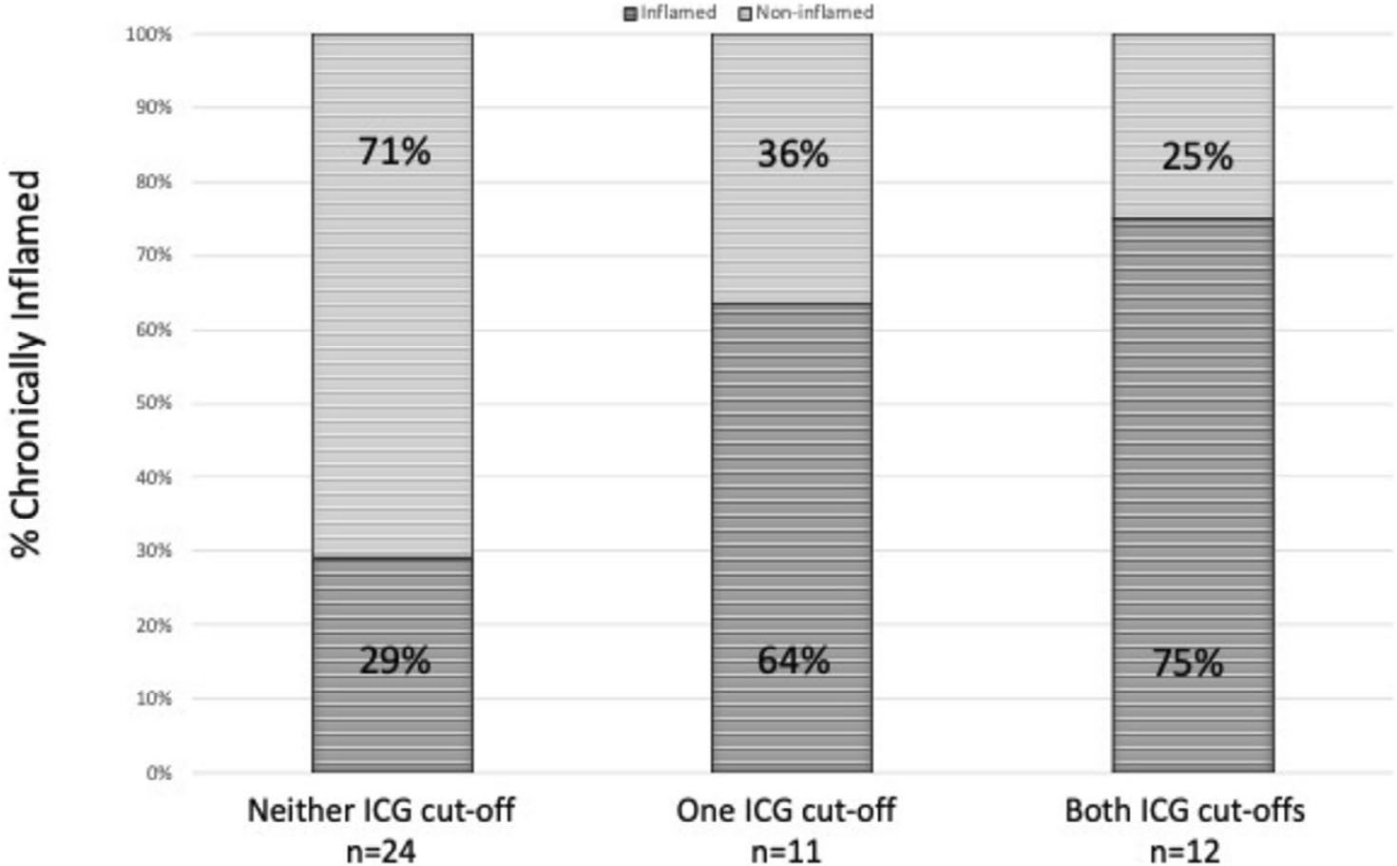
Proportion of chronically inflamed hemodialysis patients achieving indocyanine green‐plasma disappearance rate and indocyanine green‐retention after 15 min cut off values.

75% of those with both an indocyanine green‐plasma disappearance rate < 21%/min and an indocyanine green‐retention after 15 min > 7.5% were chronically inflamed, compared to 64% with either an indocyanine green‐plasma disappearance rate < 21%/min or an indocyanine green‐retention after 15 min > 7.5%, and 29% who had neither an indocyanine green‐plasma disappearance rate < 21%/min nor an indocyanine green‐retention after 15 min > 7.5% (*p* = 0.02) (Figure 3). All 12 participants who had an indocyanine green‐retention after 15 min > 7.5% also had an indocyanine green‐plasma disappearance rate < 21%/min.

### 3.3 Beta‐D‐Glucan and Fecal Calprotectin

**FIGURE 4:**
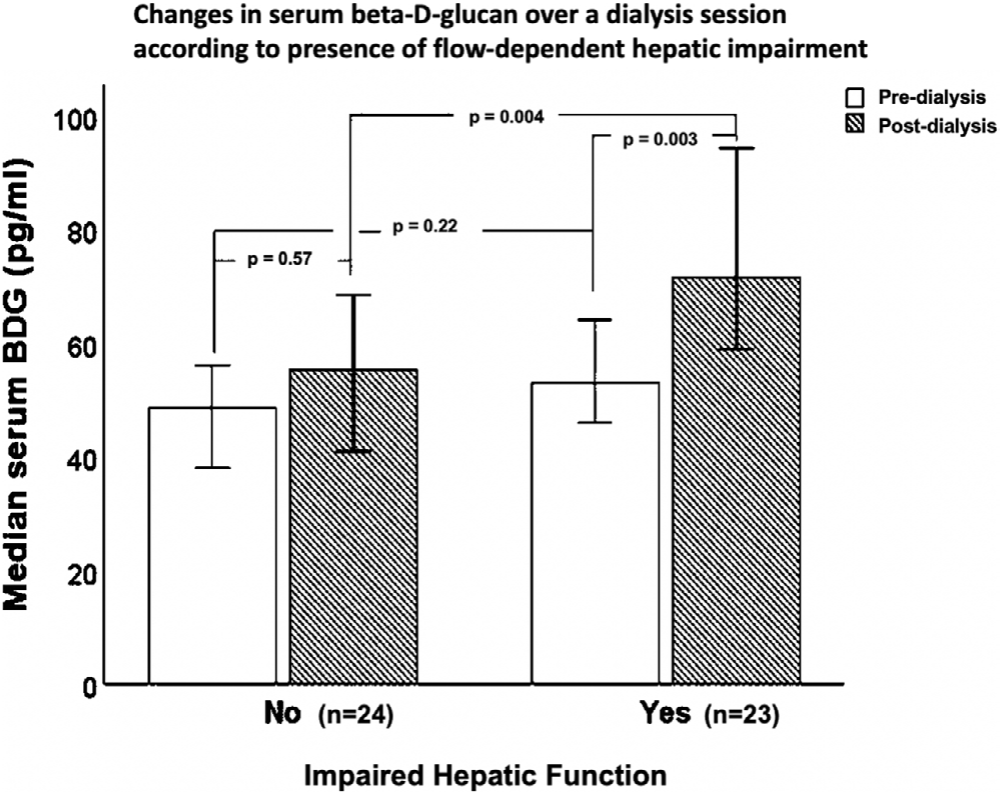
Changes in serum beta‐D‐glucan over a hemodialysis session according to the presence of flow‐dependent hepatic impairment.

Pre‐ and post‐hemodialysis beta‐D‐glucan levels taken from a single hemodialysis session along with fecal calprotectin levels are presented in Table 3. Pre‐hemodialysis beta‐D‐glucan levels were similar in both inflamed and noninflamed groups (63 (42) pg/ml vs. 49 (11) pg/ml; *p* = 0.13). Post‐hemodialysis beta‐D‐glucan levels were higher in the inflamed group (82 (49) pg/ml vs. 58 (27) pg/ml; *p* = 0.001). There was a greater rise from baseline of serum beta‐D‐glucan during a hemodialysis session in the inflamed group (24 (38) pg/ml vs. 5 (35) pg/ml; *p* = 0.02). Post‐hemodialysis beta‐D‐glucan levels were unaffected by membrane type and modality (hemodialysis vs. hemodiafiltration). However, impaired hepatic function, defined by at least one of the indocyanine green cut‐off criteria reported above, was associated with higher post‐hemodialysis beta‐D‐glucan levels (*p* = 0.004) and a greater increase of beta‐D‐glucan across the hemodialysis session (*p* = 0.003) (Figure 4). Fecal calprotectin levels were similar in both inflamed and noninflamed groups (97 (87)μg/g vs. 35 (95)μg/g; *p* = 0.19).

**TABLE 3:** Changes in beta‐D‐glucan over a hemodialysis session and baseline fecal calprotectin levels.

	Non‐inflamed (*n* = 25)	Inflamed (*n* = 25)	*p*‐value
BDG (pg/ml) (pre‐dialysis)	49 (11)	63 (42)	0.13
BDG (pg/ml) (post‐dialysis)	58 (27)	82 (49)	**0.001**
Intradialytic change in BDG (pg/ml)	5 (35)	24 (38)	**0.02**
Fecal calprotectin (μg/g)	97 (87)	35 (95)	0.19

*Note:* Results for continuous variables presented as mean ± standard deviation or median (interquartile range) according to distribution. The bold values are those that have a *p*‐value of < 0.05 and thus are deemed statistically significant.

### 3.4 Predictors of Chronic Inflammation

Median hs‐CRP correlated with post‐hemodialysis beta‐D‐glucan (rho = 0.426; *p* = 0.002), intradialytic increase in beta‐D‐glucan (rho = 0.362; *p* = 0.01), indocyanine green‐plasma disappearance rate (rho = −0.360; *p* = 0.01) and indocyanine green‐retention after 15 min (rho = 0.358; *p* = 0.01). There was no correlation with pre‐hemodialysis beta‐D‐glucan levels. The best linear regression model of Ln (median hs‐CRP) is shown in Table 4. Both Ln (post‐hemodialysis beta‐D‐glucan) and Ln (indocyanine green‐retention after 15 min) were significant in the model, which explained 21% of the variation. The best logistic regression model of the presence of chronic inflammation is shown in Table 5. Ln (post‐hemodialysis beta‐D‐glucan) was significant in the model, whilst Ln (indocyanine green‐retention after 15 min) was not. The model explained 37% of the variation.

**TABLE 4:** Linear regression model of Ln (median hs‐CRP).

Linear regression model of Ln (median hs‐CRP): adjusted *R* square = 0.211					
	*B*	Std. error	Beta	*t*	*p*
Constant	−0.561	1.653		−0.339	0.736
Ln (post‐dialysis BDG)	0.715	0.354	0.287	2.021	**0.049**
Ln (ICG‐R15)	0.304	0.304	0.304	2.319	**0.025**

*Note:* For abbreviations, see text. The bold values are those that have a *p*‐value of < 0.05 and thus are deemed statistically significant.

**TABLE 5:** Logistic Regression model of the predictors of chronic inflammation.

Logistic regression model of predictors of chronic inflammation: Nagelkerke *R* square = 0.369					
	*B*	Std. error	Wald	Exp (*B*)	*p*
Constant	−3.515	1.241	8.023	0.030	0.005
Ln (post‐dialysis BDG)	0.044	0.018	6.047	1.045	0.014
Ln (ICG‐R15)	0.095	0.064	2.216	1.100	0.137

*Note:* For abbreviations, see text.

### 3.5 Patient Reported Outcomes

Patient reported outcomes are presented in Table 6. Post‐hemodialysis recovery time (180 (703) vs. 60 (480) minutes; *p* = 0.19) and SONG‐hemodialysis fatigue scores (5 (4) vs. 3 (3); *p* = 0.19) were similar in both inflamed and noninflamed groups; as were PHQ‐8 scores (6 (7) vs. 3 (5); *p* = 0.08).

**TABLE 6:** Patient reported outcomes.

	Non‐inflamed (*n* = 25)	Inflamed (*n* = 25)	*p*
Self‐reported post dialysis recovery time (mins)	60 (480)	180 (703)	0.19
SONG‐HD fatigue score	3 (3)	5 (4)	0.19
PHQ‐8 score	3 (5)	6 (7)	0.08

*Note:* Results presented as median (interquartile range).

## 4 Discussion

This study evaluated differences in hepatic function at the end of hemodialysis between individuals with chronic inflammation and those who were noninflamed. It should be emphasized that no participants had a history of chronic liver disease, and no cause was apparent for inflammation other than kidney failure itself or its treatment (ESKD‐associated chronic inflammation). This is the first study to demonstrate impaired flow‐dependent hepatic function in chronically inflamed individuals on maintenance hemodialysis compared with noninflamed individuals. Significantly higher beta‐D‐glucan levels post‐hemodialysis and a higher intradialytic beta‐D‐glucan rise were identified in chronically inflamed individuals. Both pre‐hemodialysis serum beta‐D‐glucan and fecal calprotectin levels were higher in inflamed participants, but not significantly. These results are compatible with the theory that impaired hepatic function at the end of hemodialysis may facilitate passage of translocated gut‐derived pro‐inflammatory microbial substances from the portal to the systemic circulation. This is a plausible stimulus of chronic inflammation in ESKD.

### 4.1 Hepatic Function and Chronic Inflammation

Changes in flow‐dependent hepatic function were present despite similar markers of synthetic hepatic function and noninvasive assessment of the physical properties of hepatic tissue (steatosis and fibrosis) in both groups. Baseline demographics, underlying comorbidities, dialysis vintage and access, and volume status were similar in both groups. It was notable, however, that the chronically inflamed group in this study had a significantly greater BMI. This may reflect the relatively small number of patients recruited in this study and some of the exclusion criteria, including underlying gastrointestinal and peripheral vascular disease, which may have disproportionately limited the recruitment of wasted, inflamed individuals with lower BMI measurements. This is important in the context of previous work demonstrating greater mortality among chronically inflamed individuals, which was attenuated by higher BMI measurements [37].

Previous studies have shown that flow‐dependent hepatic function reduces both acutely, during a hemodialysis session [16, 17] and more chronically, in the setting of impaired kidney function [38]. This study builds on these findings, demonstrating that reduced hepatic function is intimately linked to chronic inflammation in ESKD. In healthy individuals, normal values for indocyanine green‐plasma disappearance rate vary between 18%–25%/min and are 0%–10% for indocyanine green‐retention after 15 min, depending on the clinical context [19, 35, 39, 40]. Given this variance, ROC analysis helped define relationships between indocyanine green‐plasma disappearance rate and indocyanine green‐retention after 15 min in inflamed individuals with ESKD. Participants with hepatic impairment, defined as an indocyanine green‐plasma disappearance rate < 21%/min and/or an indocyanine green‐retention after 15 min level > 7.5%, were significantly more likely to be chronically inflamed. The majority of noninflamed individuals had normal flow‐dependent hepatic function.

These findings suggest that abnormalities of hepatic function detected immediately following hemodialysis are associated with chronic inflammation. In order for gut‐derived toxins to enter the circulation, there first has to be translocation from the gut into the portal venous system. Following this, the toxins have to avoid removal by the liver.

The contribution of hepatic blood flow alterations to systemic inflammation in ESKD remains incompletely understood; however, it is likely that changes in hepatic perfusion are implicated, together with the delivery of blood enriched with gut‐derived microbial products. Changes in hepatic perfusion related to hemodialysis are highly complex due to the dual nature of blood supply to the liver and involve changes in hepatic arterial blood flow and an associated hepatic arterial buffer response, together with alterations in portal venous flow [9, 17, 41, 42]. The process of intravascular refilling following hemodialysis may additionally affect hepatic perfusion and, as a consequence, indocyanine green clearance in the post‐dialysis setting.

### 4.2 Beta‐D‐Glucan

Post‐hemodialysis beta‐D‐glucan levels were significantly higher in the inflamed group and also the subgroup of individuals with impaired hepatic function. The increase in intradialytic change in beta‐D‐glucan during hemodialysis was also higher in the inflamed group. Median post‐hemodialysis serum beta‐D‐glucan levels were 82 pg/mL in the inflamed group, which is above the diagnostic cut‐off value for invasive fungal infection [43]. These levels were detected in clinically stable individuals without infection. Although use of cellulose‐containing hemodialysis membranes is associated with elevated beta‐D‐glucan levels [44], this is not true of synthetic membranes, which were the predominant membrane type in this study [45]. No association between membrane type or mode of dialysis (hemodialysis or hemodiafiltration) and intradialytic change in beta‐D‐glucan was identified in this study.

Elevated post‐hemodialysis serum beta‐D‐glucan levels are compatible, therefore, with increased delivery of portal venous blood enriched with gut‐derived toxins, which may be fungal rather than bacterial in origin [46] to hepatic tissue that is poorly functional at the end of a hemodialysis session.

### 4.3 Patient Reported Outcomes

Chronic inflammation is recognized as a potential source of poor patient‐reported outcomes [47], which occur all too commonly in ESKD. In this study, there was a trend, albeit nonsignificant, toward greater burden of fatigue and depressive symptoms in those with chronic inflammation. These nonsignificant findings may reflect that this study was underpowered to examine these endpoints. Nevertheless, these outcomes may be a further indication to consider ways to address ESKD‐associated chronic inflammation.

### 4.4 Protecting Gut Barrier Integrity and Hepatic Function

Results of this study suggest that protection of both gut barrier integrity and hepatic function may help reduce the prevalence and/or severity of ESKD‐associated chronic inflammation. Adjustments to the hemodialysis prescription can improve control of the uraemic toxin levels; though little is known about the precise toxins implicated. Whether there is a role for dietary intervention is largely unexplored. Maintaining hemodynamic stability, through use of hemodiafiltration and cooled dialysate, may be desirable and have specific benefits on both gut permeability and hepatic protection; though the role of cooling has recently been called into question [48]. Despite the greater prevalence of hemodiafiltration prescription in the inflamed group (96%) versus the noninflamed group (64%), hepatic function was worse in this group. This suggests that additional interventions may be required to improve outcomes and provide hepatoprotection. The utilization of pharmacotherapy, in combination with dialytic strategies, may offer promise. Extension of the routine use of sodium‐glucose transporter 2 inhibitors and glucagon‐like peptide‐1 receptor agonists into individuals with ESKD may help provide pleiotropic effects that protect against liver injury and reduce inflammation [49, 50, 51].

### 4.5 Study Limitations

Some limitations need to be considered alongside the interpretation of these study findings. Participants were predominantly of White self‐reported ethnicity. Patients with peripheral vascular disease (a group in which chronic inflammation is highly prevalent [52]) were excluded. Both of these observations limit the generalizability of the study findings. Higher rates of obesity in the inflamed group, which could promote inflammation independently of hepatic function and gut permeability [53], were observed. The inflamed group was also more likely to be volume contracted, albeit not at a statistically significant level, compared to their noninflamed counterparts. These findings, in the context of comparable ultrafiltration rates, could theoretically induce higher rates of hemodynamic strain and associated activation of pro‐inflammatory cascades. Gamma‐glutamyl transpeptidase and alkaline phosphatase levels were higher in the inflamed group. Although noninvasive hepatic assessments were similar between groups, there is a possibility that there could be undiagnosed hepatic impairment in some cases. Additionally, the inflamed group had lower rates of residual kidney function, which could impair clearance of pro‐inflammatory cytokines, although these effects may have been offset by higher rates of hemodiafiltration [54]. The contribution of other factors to elevated post‐hemodialysis serum beta‐D‐glucan, including BMI, residual kidney function, and hemodiafiltration treatment (all of which were significantly different between inflamed and noninflamed groups) requires further evaluation.

This is an early‐phase observational study and consequently results are hypothesis generating. Nevertheless, results of this study can inform further studies to investigate the longer‐term effects of hepatic impairment on outcomes and interventional studies to provide hepatoprotection and minimize ESKD‐associated chronic inflammation.

## 5 Conclusions

In conclusion, flow‐dependent hepatic function, measured following a hemodialysis session, was intimately linked to ESKD‐associated chronic inflammation. Individuals with chronic inflammation exhibited higher serum beta‐D‐glucan levels post‐hemodialysis and greater intradialytic increases in beta‐D‐glucan. These findings are compatible with the theory that impaired hepatic removal of gut‐derived microbial material may be a key propagator of ESKD‐associated chronic inflammation.

## Conflicts of Interest

M.A.F. was an employee of Associates of Cape Cod Inc., who manufactures the Fungitell assay used in this study to measure beta‐D‐glucan during the study period. Y.Z. is currently employed by Associates of Cape Cod Inc.

## Data Availability

The data that support the findings of this study are available from the corresponding author upon reasonable request.

## References

[1] P. Stenvinkel , O. Heimbürger , F. Paultre , et al., “Strong Association Between Malnutrition, Inflammation, and Atherosclerosis in Chronic Renal Failure,” Kidney International 55, no. 5 (1999): 1899–1911. 10231453 10.1046/j.1523-1755.1999.00422.x

[2] K. Kalantar‐Zadeh , T. A. Ikizler , G. Block , M. M. Avram , and J. D. Kopple , “Malnutrition‐Inflammation Complex Syndrome in Dialysis Patients: Causes and Consequences,” American Journal of Kidney Diseases 42, no. 5 (2003): 864–881. 14582032 10.1016/j.ajkd.2003.07.016

[3] J. Dou , H. Liu , Y. Ma , Y. Y. Wu , and X. B. Tao , “Prevalence of Post‐Dialysis Fatigue: A Systematic Review and Meta‐Analysis,” BMJ Open 13, no. 6 (2023): e064174. 10.1136/bmjopen-2022-064174 PMC10277119 37311633

[4] G. Cobo , B. Lindholm , and P. Stenvinkel , “Chronic Inflammation in End‐Stage Renal Disease and Dialysis,” Nephrology, Dialysis, Transplantation 33, no. suppl_3 (2018): iii35–iii40. 10.1093/ndt/gfy175 PMC6168801 30281126

[5] F. Wang , H. Jiang , K. Shi , Y. Ren , P. Zhang , and S. Cheng , “Gut Bacterial Translocation Is Associated With Microinflammation in End‐Stage Renal Disease Patients,” Nephrology 17, no. 8 (2012): 733–738. 22817644 10.1111/j.1440-1797.2012.01647.x

[6] N. D. Vaziri , J. Yuan , and K. Norris , “Role of Urea in Intestinal Barrier Dysfunction and Disruption of Epithelial Tight Junction in Chronic Kidney Disease,” American Journal of Nephrology 37, no. 1 (2013): 1–6. 23258127 10.1159/000345969 PMC3686571

[7] N. Bassilios , V. Menoyo , A. Berger , et al., “Mesenteric Ischaemia in Hemodialysis Patients: A Case/Control Study,” Nephrology, Dialysis, Transplantation 18, no. 5 (2003): 911–917. 10.1093/ndt/gfg004 12686664

[8] C. J. Grant , S.‐H. S. Huang , and C. W. McIntyre , “Hepato‐Splanchnic Circulatory Stress: An Important Effect of Hemodialysis,” Seminars in Dialysis 32, no. 3 (2019): 237–242. 30937954 10.1111/sdi.12782

[9] J. T. Daugirdas , “Intradialytic Hypotension and Splanchnic Shifting: Integrating an Overlooked Mechanism With the Detection of Ischemia‐Related Signals During Hemodialysis,” Seminars in Dialysis 32, no. 3 (2019): 243–247. 30864293 10.1111/sdi.12781

[10] J. Luther , J. J. Garber , H. Khalili , et al., “Hepatic Injury in Nonalcoholic Steatohepatitis Contributes to Altered Intestinal Permeability,” Cellular and Molecular Gastroenterology and Hepatology 1, no. 2 (2015): 222–232. 26405687 10.1016/j.jcmgh.2015.01.001 PMC4578658

[11] E. Papachristoforou and P. Ramachandran , “Macrophages as Key Regulators of Liver Health and Disease,” International Review of Cell and Molecular Biology 368 (2022): 143–212. 35636927 10.1016/bs.ircmb.2022.04.006

[12] O. Swift , E. Vilar , and K. Farrington , “Unexplained Inflammation in End‐Stage Kidney Disease: Is the Combination of Enhanced Gastrointestinal Permeability and Reticuloendothelial Dysfunction Its Cause?,” Seminars in Dialysis 32, no. 5 (2019): 417–423. 30968463 10.1111/sdi.12810

[13] C. C. Chien , J. J. Wang , Y. M. Sun , et al., “Long‐Term Survival and Predictors for Mortality Among Dialysis Patients in an Endemic Area for Chronic Liver Disease: A National Cohort Study in Taiwan,” BMC Nephrology 13 (2012): 43. 22709415 10.1186/1471-2369-13-43 PMC3422197

[14] A. J. Kim , H. J. Lim , H. Ro , et al., “Liver Cirrhosis Leads to Poorer Survival in Patients With End‐Stage Renal Disease,” Korean Journal of Internal Medicine 31, no. 4 (2016): 730–738. 27017394 10.3904/kjim.2014.328 PMC4939491

[15] O. Swift , S. Sharma , S. Ramanarayanan , et al., “Prevalence and Outcomes of Chronic Liver Disease in Patients Receiving Dialysis: Systematic Review and Meta‐Analysis,” Clinical Kidney Journal 15, no. 4 (2022): 747–757. 35371444 10.1093/ckj/sfab230 PMC8967682

[16] W. Ribitsch , D. Schneditz , C. F. Franssen , et al., “Increased Hepato‐Splanchnic Vasoconstriction in Diabetics During Regular Hemodialysis,” PLoS One 10, no. 12 (2015): e0145411. 26713734 10.1371/journal.pone.0145411 PMC4695079

[17] R. Marants , E. Qirjazi , K. B. Lai , et al., “Exploring the Link Between Hepatic Perfusion and Endotoxemia in Hemodialysis,” Kidney International Reports 6, no. 5 (2021): 1336–1345. 34013112 10.1016/j.ekir.2021.02.008 PMC8116762

[18] G. R. Cherrick , S. W. Stein , C. M. Leevy , and C. S. Davidson , “Indocyanine Green: Observations on Its Physical Properties, Plasma Decay, and Hepatic Extraction,” Journal of Clinical Investigation 39, no. 4 (1960): 592–600. 13809697 10.1172/JCI104072 PMC293343

[19] P. Faybik and H. Hetz , “Plasma Disappearance Rate of Indocyanine Green in Liver Dysfunction,” Transplantation Proceedings 38, no. 3 (2006): 801–802. 16647475 10.1016/j.transproceed.2006.01.049

[20] A. Zipprich , O. Kuss , S. Rogowski , et al., “Incorporating Indocyanin Green Clearance Into the Model for End Stage Liver Disease (MELD‐Indocyanine Green) Improves Prognostic Accuracy in Intermediate to Advanced Cirrhosis,” Gut 59, no. 7 (2010): 963–968. 20581243 10.1136/gut.2010.208595

[21] B. M. Halle , T. D. Poulsen , and H. P. Pedersen , “Indocyanine Green Plasma Disappearance Rate as Dynamic Liver Function Test in Critically Ill Patients,” Acta Anaesthesiologica Scandinavica 58, no. 10 (2014): 1214–1219. 25307706 10.1111/aas.12406

[22] J. Wong , K. Lenaerts , D. M. Meesters , et al., “Acute Haemodynamic Changes During Hemodialysis Do Not Exacerbate Gut Hyperpermeability,” Bioscience Reports 39, no. 4 (2019): BSR20181704. 30898976 10.1042/BSR20181704 PMC6477914

[23] M. Hoenigl , J. Pérez‐Santiago , M. Nakazawa , et al., “(1→3)‐β‐d‐Glucan: A Biomarker for Microbial Translocation in Individuals With Acute or Early HIV Infection?,” Frontiers in Immunology 7 (2016): 404. 27752257 10.3389/fimmu.2016.00404 PMC5046804

[24] P. J. Rice , E. L. Adams , T. Ozment‐Skelton , et al., “Oral Delivery and Gastrointestinal Absorption of Soluble Glucans Stimulate Increased Resistance to Infectious Challenge,” Journal of Pharmacology and Experimental Therapeutics 314, no. 3 (2005): 1079–1086. 15976018 10.1124/jpet.105.085415

[25] N. N. Miura , N. Ohno , J. Aketagawa , H. Tamura , S. Tanaka , and T. Yadomae , “Blood Clearance of (1→3)‐Beta‐D‐Glucan in MRL Lpr/Lpr Mice,” FEMS Immunology and Medical Microbiology 13, no. 1 (1996): 51–57. 8821398 10.1111/j.1574-695X.1996.tb00215.x

[26] J. Wong , Y. Zhang , O. Swift , et al., “Beta‐Glucans in Advanced CKD: Role in Endotoxaemia and Inflammation,” BMC Nephrology 21, no. 1 (2020): 118. 32252666 10.1186/s12882-020-01779-9 PMC7137517

[27] J. Wong , Y. Zhang , A. Patidar , E. Vilar , M. Finkelman , and K. Farrington , “Is Endotoxemia in Stable Hemodialysis Patients an Artefact? Limitations of the Limulus Amebocyte Lysate Assay and Role of (1→3)‐β‐D Glucan,” PLoS One 11, no. 10 (2016): e0164978. 27764208 10.1371/journal.pone.0164978 PMC5072723

[28] V. Panichi , G. M. Rizza , D. Taccola , et al., “C‐Reactive Protein in Patients on Chronic Hemodialysis With Different Techniques and Different Membranes,” Biomedicine and Pharmacotherapy 60, no. 1 (2006): 14–17. 16330177 10.1016/j.biopha.2005.06.013

[29] V. Panichi , G. M. Rizza , S. Paoletti , et al., “Chronic Inflammation and Mortality in Haemodialysis: Effect of Different Renal Replacement Therapies. Results From the RISCAVID Study,” Nephrology, Dialysis, Transplantation 23, no. 7 (2008): 2337–2343. 10.1093/ndt/gfm951 18305316

[30] A. Ju , A. Teixeira‐Pinto , A. Tong , et al., “Validation of a Core Patient‐Reported Outcome Measure for Fatigue in Patients Receiving Hemodialysis: The SONG‐Hemodialysis Fatigue Instrument,” Clinical Journal of the American Society of Nephrology 15, no. 11 (2020): 1614–1621. 33093215 10.2215/CJN.05880420 PMC7646231

[31] K. Kroenke , T. W. Strine , R. L. Spitzer , J. B. Williams , J. T. Berry , and A. H. Mokdad , “The PHQ‐8 as a Measure of Current Depression in the General Population,” Journal of Affective Disorders 114, no. 163 (2009): 163–173. 18752852 10.1016/j.jad.2008.06.026

[32] R. Petraitiene , V. Petraitis , W. W. Hope , et al., “Cerebrospinal Fluid and Plasma (1→3)‐β‐d‐Glucan as Surrogate Markers for Detection and Monitoring of Therapeutic Response in Experimental HematogenousCandidaMeningoencephalitis,” Antimicrobial Agents and Chemotherapy 52, no. 11 (2008): 4121–4129. 18779361 10.1128/AAC.00674-08 PMC2573149

[33] M. Tabinor and S. J. Davies , “The Use of Bioimpedance Spectroscopy to Guide Fluid Management in Patients Receiving Dialysis,” Current Opinion in Nephrology and Hypertension 27, no. 6 (2018): 406–412. 30063488 10.1097/MNH.0000000000000445

[34] S. G. Sakka and N. van Hout , “Relation Between Indocyanine Green (Indocyanine Green) Plasma Disappearance Rate and Indocyanine Green Blood Clearance in Critically Ill Patients,” Intensive Care Medicine 32, no. 5 (2006): 766–769. 16544120 10.1007/s00134-006-0109-6

[35] T. Imai , K. Takahashi , F. Goto , and Y. Morishita , “Measurement of Blood Concentration of Indocyanine Green by Pulse Dye Densitometry—Comparison With the Conventional Spectrophotometric Method,” Journal of Clinical Monitoring and Computing 14, no. 7–8 (1998): 477–484. 10385856 10.1023/a:1009948128543

[36] R. K. Sterling , E. Lissen , N. Clumeck , et al., “Development of a Simple Noninvasive Index to Predict Significant Fibrosis in Patients With HIV/HCV Coinfection,” Hepatology 43, no. 6 (2006): 1317–1325. 16729309 10.1002/hep.21178

[37] P. Stenvinkel , I. A. Gillespie , J. Tunks , et al., “Inflammation Modifies the Paradoxical Association Between Body Mass Index and Mortality in Hemodialysis Patients,” Journal of the American Society of Nephrology 27, no. 5 (2016): 1479–1486. 26567245 10.1681/ASN.2015030252 PMC4849822

[38] A. Tokunaga , H. Miyamoto , S. Fumoto , and K. Nishida , “Effect of Chronic Kidney Disease on Hepatic Clearance of Drugs in Rats,” Biological and Pharmaceutical Bulletin 43, no. 9 (2020): 1324–1330. 32879206 10.1248/bpb.b20-00124

[39] L. B. Rowell , J. R. Blackmon , and R. A. Bruce , “Indocyanine Green Clearance and Estimated Hepatic Blood Flow During Mild to Maximal Exercise in Upright Man,” Journal of Clinical Investigation 43, no. 8 (1964): 1677–1690. 14201551 10.1172/JCI105043 PMC441967

[40] A. De Gasperi , E. Mazza , and M. Prosperi , “Indocyanine Green Kinetics to Assess Liver Function: Ready for a Clinical Dynamic Assessment in Major Liver Surgery?,” World Journal of Hepatology 8, no. 7 (2016): 355–367. 26981173 10.4254/wjh.v8.i7.355 PMC4779164

[41] C. W. McIntyre , “Update on Hemodialysis‐Induced Multiorgan Ischemia: Brains and Beyond,” Journal of the American Society of Nephrology 35, no. 5 (2024): 653–664. 38273436 10.1681/ASN.0000000000000299 PMC11149050

[42] S. M. Jakob , “Clinical Review: Splanchnic Ischaemia,” Critical Care 6 (2002): 306–312. 12225604 10.1186/cc1515 PMC137310

[43] Z. Odabasi , G. Mattiuzzi , E. Estey , et al., “Beta‐D‐Glucan as a Diagnostic Adjunct for Invasive Fungal Infections: Validation, Cutoff Development, and Performance in Patients With Acute Myelogenous Leukemia and Myelodysplastic Syndrome,” Clinical Infectious Diseases 39, no. 2 (2004): 199–205. 15307029 10.1086/421944

[44] F. C. Pearson , J. Bohon , W. Lee , et al., “Characterization of Limulus Amoebocyte Lysate‐Reactive Material From Hollow‐Fiber Dialyzers,” Applied and Environmental Microbiology 48, no. 6 (1984): 1189–1196. 6517586 10.1128/aem.48.6.1189-1196.1984 PMC241708

[45] J. Prattes , D. Schneditz , F. Prüller , et al., “1,3‐ß‐d‐Glucan Testing Is Highly Specific in Patients Undergoing Dialysis Treatment,” Journal of Infection 74, no. 1 (2017): 72–80. 27717781 10.1016/j.jinf.2016.09.005

[46] P. A. Rootjes , M. P. C. Grooteman , A. E. Budding , et al., “Randomized Trial Demonstrating no Translocation of Intact Intestinal Bacteria During Hemodialysis or Hemodiafiltration,” Kidney International Reports 10, no. 1 (2024): 109–119. 39810793 10.1016/j.ekir.2024.09.025 PMC11725968

[47] A. D. H. Brys , E. Di Stasio , B. Lenaert , et al., “Serum Interleukin‐6 and Endotoxin Levels and Their Relationship With Fatigue and Depressive Symptoms in Patients on Chronic Hemodialysis,” Cytokine 125 (2020): 154823. 31541903 10.1016/j.cyto.2019.154823

[48] A. X. Garg , A. A. Al‐Jaishi , and S. N. Dixon , “Personalised Cooler Dialysate for Patients Receiving Maintenance Hemodialysis (MyTEMP): A Pragmatic, Cluster‐Randomised Trial,” Lancet 400, no. 10364 (2022): 1693–1703. 36343653 10.1016/S0140-6736(22)01805-0

[49] C. Fang , J. Pan , N. Qu , et al., “The AMPK Pathway in Fatty Liver Disease,” Frontiers in Physiology 13 (2022): 970292. 36203933 10.3389/fphys.2022.970292 PMC9531345

[50] N. Safaie , S. Masoumi , S. Alizadeh , et al., “SGLT2 Inhibitors and AMPK: The Road to Cellular Housekeeping?,” Cell Biochemistry and Function 42, no. 1 (2024): e3922. 38269506 10.1002/cbf.3922

[51] M. P. Valdecantos , L. Ruiz , V. Pardo , et al., “Differential Effects of a Glucagon‐Like Peptide 1 Receptor Agonist in Non‐Alcoholic Fatty Liver Disease and in Response to Hepatectomy,” Scientific Reports 8, no. 1 (2018): 16461. 30405191 10.1038/s41598-018-33949-z PMC6220318

[52] Á. G. Rojas , A. V. Martínez , P. R. Benítez , et al., “Peripheral Arterial Disease in Hemodialysis Patients 10 Years Later,” Nefrología 43, no. 3 (2023): 302–308. 37625979 10.1016/j.nefroe.2022.01.014

[53] T. Kawai , M. V. Autieri , and R. Scalia , “Adipose Tissue Inflammation and Metabolic Dysfunction in Obesity,” American Journal of Physiology. Cell Physiology 320, no. 3 (2021): C375–c391. 33356944 10.1152/ajpcell.00379.2020 PMC8294624

[54] M. Wolley , M. Jardine , and C. A. Hutchison , “Exploring the Clinical Relevance of Providing Increased Removal of Large Middle Molecules,” Clinical Journal of the American Society of Nephrology 13, no. 5 (2018): 805–814. 29507008 10.2215/CJN.10110917 PMC5969479

